# AMPEC4: *Naja ashei* Venom-Derived Peptide as a Stimulator of Fibroblast Migration with Antibacterial Activity

**DOI:** 10.3390/molecules30102167

**Published:** 2025-05-15

**Authors:** Ewa Ciszkowicz, Anna Miłoś, Andrzej Łyskowski, Justyna Buczkowicz, Anna Nieczaj, Katarzyna Lecka-Szlachta, Konrad K. Hus, Karol Sikora, Damian Neubauer, Marta Bauer, Wojciech Kamysz, Aleksandra Bocian

**Affiliations:** 1 Department of Biotechnology and Bioinformatics, Faculty of Chemistry, Rzeszów University of Technology, al. Powstańców Warszawy 6, 35-959 Rzeszów, Poland; a.milos@prz.edu.pl (A.M.); czaporj@prz.edu.pl (J.B.); szlachta@prz.edu.pl (K.L.-S.); k.hus@prz.edu.pl (K.K.H.); bocian@prz.edu.pl (A.B.); 2 Doctoral School of the Rzeszów University of Technology, al. Powstańców Warszawy 12, 35-959 Rzeszów, Poland; a.nieczaj@prz.edu.pl; 3 Department of Inorganic Chemistry, Faculty of Pharmacy, Medical University of Gdańsk, al. Gen. J. Hallera 107, 80-416 Gdańsk, Poland; karol.sikora@gumed.edu.pl (K.S.); damian.neubauer@gumed.edu.pl (D.N.); wojciech.kamysz@gumed.edu.pl (W.K.); 4 Department of Analytical Chemistry, Faculty of Pharmacy, Medical University of Gdańsk, al. Gen. J. Hallera 107, 80-416 Gdańsk, Poland; marta.bauer@gumed.edu.pl

**Keywords:** antimicrobial peptide, antibiofilm activity, *Naja ashei*, *Escherichia coli*, wound healing, fibroblasts, proctology

## Abstract

The treatment of proctological conditions, including hemorrhoids, anal fissures, and perianal abscesses, is often complicated by bacterial infections, particularly those involving multidrug-resistant *Escherichia coli*. This study presents the synthesis, characterization, and biological evaluation of the newly designed synthetic peptide AMPEC4, inspired by cytotoxin 5 from *Naja ashei* snake venom. AMPEC4 demonstrated potent antimicrobial properties with MIC values of 100 and 200 µg/mL, effectively inhibiting biofilm formation (up to 84%) and eradicating the pre-formed biofilm by up to 35%. The antibacterial activity of AMPEC4 was further supported by a membrane permeabilization assay, demonstrating its capacity to disrupt bacterial membrane integrity in a dose-dependent manner. Furthermore, AMPEC4 significantly promoted fibroblast migration, a critical step in tissue regeneration, while exhibiting notable biocompatibility, as evidenced by the absence of hemolytic, cytotoxic, and genotoxic effects. By addressing both infection control and tissue regeneration, AMPEC4 represents a promising therapeutic strategy for managing chronic wounds, particularly in the challenging environment of the anorectal region. Its ability to target *Escherichia coli* reference and clinical strains while accelerating the wound-healing process underscores its potential for future clinical applications.

## 1. Introduction

Snake venoms have long served as a valuable resource for the design of novel pharmacologically active compounds, and their utility as structural scaffolds for therapeutic peptide engineering has garnered significant attention [1]. This complex mixture of biological components comprises a diverse array of proteinaceous constituents, encompassing both catalytic entities, such as metalloproteases, serine proteases, and phospholipases A_2_, and non-enzymatic components, including three-finger toxins (3FTxs), cysteine-rich secretory proteins (CRISPs), and bioactive peptides like cathelicidins and bradykinin-potentiating peptides [2]. Leveraging components from snake venoms with known properties represents a valuable approach for the development of novel bioactive compounds.

Despite the increasing success of peptide-based drugs, representing over 7% of recent FDA approvals [3,4], their design and optimization remain significant challenges [5,6], prompting the development of various predictive models and tools [7,8]. Nevertheless, there are many scientific reports on the synthesis of peptides based on the components of snake venoms, a large group of which are those with dual biological properties [4,6,9,10]. Many peptides, based on phospholipase A_2_ (PLA_2_) sequence, revealed activity against various pathogens, including Gram-positive (pBmTxJ [6], p-AppK and p-Acl [4]) and Gram-negative (pCergo [6], p-AppK and p-Acl [4]) bacteria, a parasite (pCergo [6]), and cancer cells (pBmje [6], p-AppK and p-Acl [4]). Another example is peptide LZ1, designed on the basis of snake venom cathelicidin, which significantly inhibited the growth of pancreatic cancer [11].

Cytotoxin 5 is an approximately 7 kDa polypeptide, comprising 60 amino acid residues, that adopts a canonical 3FTx fold. Functionally, it is classified as a cardio- and cytotoxin, exhibiting a mechanism of action predicated on phospholipid binding and subsequent cytolysis via pore formation in lipid bilayers [12,13]. Cardiotoxins, including cytotoxin 5, derived from the venoms of *Naja nigricollis* [14] and *Naja naja atra* [15], have been recognized as effective antibacterial agents against Gram-positive and Gram-negative bacteria.

The anorectal region, characterized by its unique microflora and intricate anatomical structure, is particularly vulnerable to bacterial infections and tissue damage [16,17]. Proctological conditions such as hemorrhoids, anal fissures, fistulas, and abscesses often disrupt the integrity of the mucosal and skin barriers, creating an environment conducive to pathogen colonization [18]. These prevalent conditions affect a substantial portion of the population. Hemorrhoids alone are estimated to affect approximately 50% of adults by the age of 50, highlighting the need for effective treatment options [19]. Perianal abscesses and anal pruritus, as hemorrhoids symptoms, are significantly associated with the proliferation of pathogenic bacteria in the perianal region, especially *Escherichia coli*, *Klebsiella pneumoniae*, and *Staphylococcus aureus* [18,19,20,21]. In anal abscesses and fistulas, *E. coli* strains were found as the major microorganisms [18,20,21], and high rates of resistances against everyday antibiotics (e.g., penicillin, fluoroquinolones, first and second generation cephalosporins), including perioperative antibiotic prophylaxis, were alarming [20]. Additionally, *E. coli* isolated from anal fissures formed a thick biofilm, which in 100% of cases complicates the antimicrobial therapy and defines its chronic development [20,22]. Consequently, the effective management of these proctological conditions requires not only the eradication of infections caused by multidrug-resistant bacteria but also the enhancement of tissue regeneration processes [23].

Wound healing is a complex biological process that involves several stages, including hemostasis, inflammation, proliferation, and remodeling [24]. A critical aspect of the proliferative phase is the migration of fibroblasts, which is essential for tissue repair, collagen synthesis, and modification [25,26], as well as secretion of various growth factors [27]. The stimulation of fibroblast migration by bioactive peptides can significantly enhance the wound healing process, making them valuable candidates for therapeutic applications in both acute and chronic wounds. The ability of wound-healing peptides (WHPs) to promote cellular migration and proliferation has been well documented, thus, they can serve as an effective supplementation or even replacement for traditional ingredients, e.g., hyaluronic acid [28] or chitosan [29].

This study focuses on developing novel synthetic peptides that exhibit antibacterial properties and the capacity to accelerate fibroblast migration. This peptide was designed to mimic the attributes of natural antimicrobial peptides (AMPs), offering a targeted approach to combating infections.

## 2. Results

### 2.1. AMPEC4 Structural Characterization

The peptide AMPEC4, with the sequence LKCKKLIPLFSKTCP-NH_2_, was designed based on a tryptic peptide from cytotoxin 5 (P25517) from the venom of *Naja mossambica*. An analog of this 3FTx was identified during the analysis of fraction 2 from *Naja ashei* venom, obtained through ion-exchange chromatography [30]. The original peptide sequence, LKCKKLIPLFSKTCPEGK, was modified by removing three C-terminal amino acids, resulting in a peptide with a mass of 1718.25 Da, a +4 charge, and hydrophobicity ranging from 33% [31] to 47% [32], as determined by different calculation methods. The peptide also exhibited a Grand Average Hydropathy (GRAVY) value of 0.23, a Protein-binding Potential (Boman index) of 0.19 kcal/mol, and a Wimley–White hydrophobicity value of 1.53 [32].

**Figure 1:**
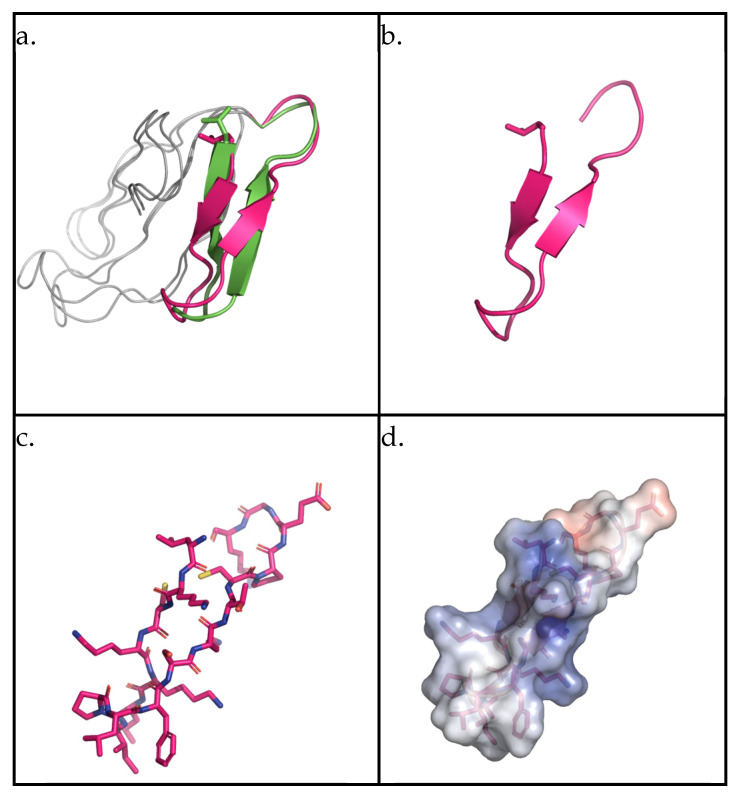
Structural representation of identified proteins containing the AMPEC4 domain. (**a**) The P25517 AMPEC4 domain is presented by magenta, P01441 by green, and the remaining portion of the proteins by gray. (**b**) The AMPEC4 domain based on the P25517 represented by a cartoon. (**c**) The AMPEC4 domain structure represented by sticks. (**d**) The electrostatic surface representation for AMPEC4 domain calculated by APBS Electrostatic PyMOL plugin for P25517 domain. The figure was prepared with PyMol open-source version 3.1.0.

The secondary structure of the AMPEC4 peptide was evaluated based on available domain models. For that purpose, a BLAST search (https://www.uniprot.org/, access date 18 March 2025) against the UniProt database was performed. A total of six hits with sequence identity above 90% were identified. In the case of P25517, identity was 100%; however, only homology models were available for this entry. For P01441, showing 93.3% sequence identity, experimental models were available (PDB ID: 1CB9). A comparison of the overall similarity of the models and the domain representing AMPEC4 shows a high degree of similarity (Figure 1a). The AMPEC4 domain is represented by two antiparallel *β*-sheets splitting the peptide roughly in half. The calculated electrostatic map shows polarization with positive and negative charges located at the opposite ends.

### 2.2. AMPEC4 with Anti-Escherichia coli Activity

#### 2.2.1. Minimum Inhibitory Concentration (MIC)

The antibacterial properties of the tested peptide against *Escherichia coli* were evaluated using the serial microdilution method [30,33]. This approach allowed for the determination of the minimal inhibitory concentration (MIC), providing insight into the peptide’s effectiveness in inhibiting the growth of both certified and clinical bacterial strains. The results obtained demonstrate antibacterial activity of AMPEC4 against the reference *E. coli* (ATCC 10536, MIC = 100 µg/mL) and clinical *E. coli* strains (MIC = 100 µg/mL or MIC = 200 µg/mL) (Table 1). Interestingly, ampicillin was ineffective against the *E. coli* 665 and 672 clinical strains, while AMPEC4 showed activity in inhibiting the growth of these strains at levels of 100 and 200 µg/mL, respectively. This underscores the high potential of the synthesized peptide as a promising antimicrobial agent.

#### 2.2.2. Inhibition of Biofilm Formation by AMPEC4

**Figure 2:**
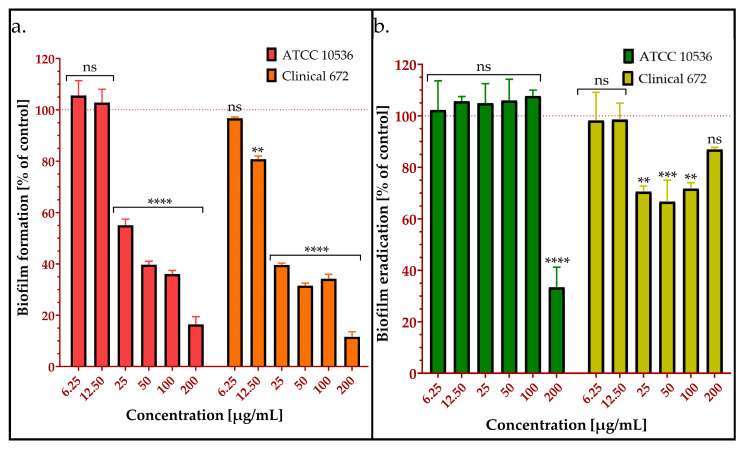
Anti-biofilm activity (**a**) and ability to eradicate the pre-formed biofilm (**b**) of AMPEC4 against reference *Escherichia coli* ATCC 10536 and clinical *E. coli* 672 strains. The statistical significance between groups treated with different concentrations of AMPEC4 and non-treated control was evaluated using the one-way ANOVA test followed by Dunnett’s multiple comparisons (** *p* < 0.005, *** *p* < 0.0005, **** *p* < 0.0001, no statistical significance compared to untreated control (ns)).

The ability of the synthetic peptide to inhibit *E. coli* biofilm formation was assessed to evaluate its potential anti-biofilm activity. This analysis provides insight into the peptide’s effectiveness in preventing bacterial adherence and biofilm development, which are crucial factors in persistent infections [20]. The results indicated that even at a concentration of ¼ MIC (25 µg/mL), AMPEC4 inhibited biofilm formation by more than 49% compared to the untreated control (Figure 2a). Biofilm formation, expressed as a percentage relative to the control, was reduced to 16%, 64%, and 60% in the presence of AMPEC4 at concentrations of 200, 100, and 50 µg/mL, respectively. Among the three clinical *E. coli* strains tested, only *E. coli* 672 formed a biofilm, whereas *E. coli* 325 and *E. coli* 665 did not exhibit biofilm formation, so these two strains were excluded from this experiment.

#### 2.2.3. Biofilm Eradication by AMPEC4

Bacterial biofilms are structured communities encased in a polymeric matrix (65–95% of total biofilm volume), adhering to surfaces. Biofilm-associated bacteria exhibit significantly enhanced antimicrobial tolerance (up to 1000-fold) compared to their planktonic counterparts [34], conferring a survival advantage. Thus, research on bacterial biofilm removal is critical for addressing the significant challenges posed by biofilm-associated infections across various fields, ultimately aiming to improve wound healing by developing novel therapeutics. The ability to remove biofilm has been tested on reference *E. coli* ATCC 10536 and clinical biofilm-forming *E. coli* 672 (Figure 2b). The results show that at low concentrations, AMPEC4 does not eradicate pre-formed biofilm, up to 100 µg/mL and 12.5 µg/mL, respectively, by the reference and clinical strain. However, at higher concentrations, biofilm is effectively eliminated in 65% (*E. coli* ATCC 10536) and in approx. 30% (*E. coli* 672).

#### 2.2.4. AMPEC4 Increase *E. coli* Membrane Permeability

**Figure 3:**
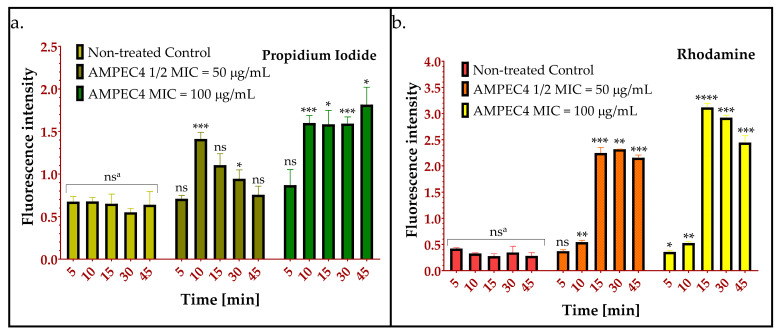
*Escherichia coli* ATCC 10536 membrane penetration of PI (**a**) and rhdAMPEC4 (**b**) after incubation with AMPEC4-rhodamine-labeled peptide in ½ MIC and MIC concentrations; ^a^—no significant differences between 5, 10, 15, 30 and 45 min of incubation of untreated control; statistical significance between groups treated with the appropriate AMPEC4 peptide concentration (½ MIC and MIC) and untreated control after the same incubation time: * *p* < 0.05, ** *p* < 0.005, *** *p* < 0.0005, **** *p* < 0.0001, ns—no statistical significance compared to untreated control.

To assess the capacity of peptide to enhance cell membrane permeability, the rhodamine-labeled AMPEC4 peptide (rhdAMPEC4) and the membrane-impermeable DNA-binding dye propidium iodide (PI) were utilized. This approach is widely used, as PI can only enter cells with compromised bacterial membranes, serving as a well-established marker of membrane integrity [35,36]. RhdAMPEC4 was used to assess its penetration through the bacterial membrane and to evaluate potential leakage from the cell due to increased membrane permeability. It is evident that AMPEC4 induces *E. coli* membrane disruption and permeability changes become irreversible after treatment with 100 µg/mL (Figure 3a). A peak in propidium iodide (PI) uptake is observed after 10 min of incubation with ½ MIC of AMPEC4. Subsequently, a decrease in fluorescence intensity over time indicates bacterial recovery after 50 µg/mL AMPEC4, with no significant differences observed at 45 min of incubation compared to the untreated control. These findings are supported by rhdAMPEC4 treatment (Figure 3b), where peptide influx remains sustained even after 45 min of incubation with 50 µg/mL rhdAMPEC4. Additionally, a decrease in fluorescence intensity is observed starting at 30 min post-treatment with 100 µg/mL rhdAMPEC4. This suggests a disruption of membrane integrity as a consequence of incubation with rhdAMPEC4 at the MIC.

### 2.3. AMPEC4 Promotes the Migration of Fibroblasts

**Figure 4:**
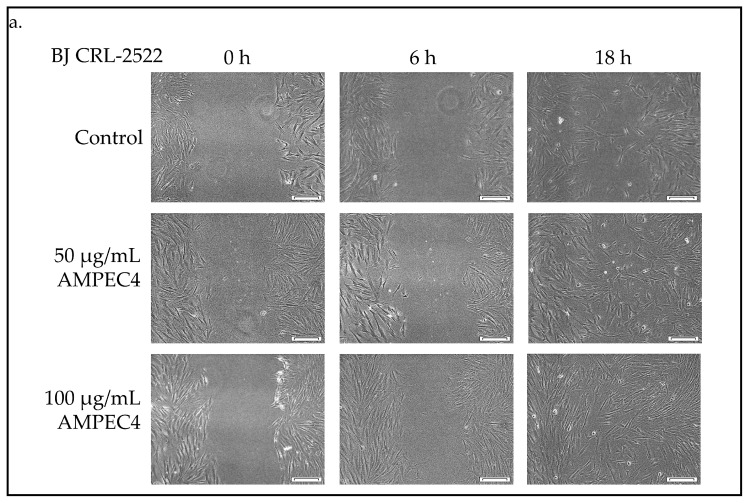

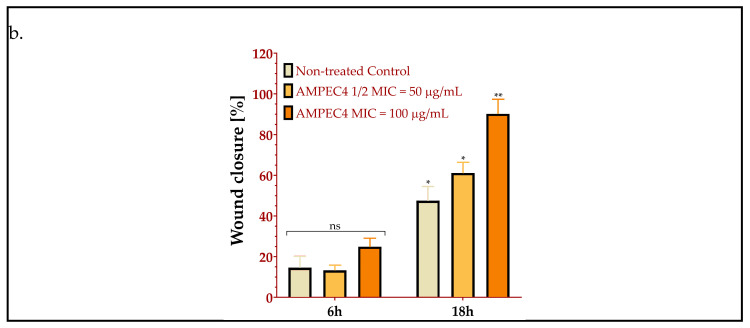
AMPEC4 promotes the migration of fibroblasts observed after 6 and 18 h of incubation in EMEM with 5% FBS; scale bar—100 µm (**a**). Statistical significance between groups treated with the appropriate AMPEC4 peptide concentration (½ MIC and MIC) and untreated control after the same incubation time (6 and 18 h): * *p* < 0.05, ** *p* < 0.005, no statistical significance (ns) (**b**).

The wound healing potential of the samples was assessed using a scratch assay on the BJ cell line. Cellular migration in response to ½ MIC and MIC concentrations of AMPEC4 was compared to the untreated control. Microscopic images were utilized to monitor the closure of the induced gap within the confluent cell monolayer (Figure 4a). The quantification of the cell-free area was performed using ImageJ software, and the data are presented as mean ± standard error (SE). AMPEC4 exhibited the strongest promigratory effects in BJ cells, with wound closure values of 61% and 90%, respectively, for ½ MIC and MIC concentrations after 18 h (Figure 4b). A 50 µg/mL measure of AMPEC4 promotes fibroblast migration compared to the untreated control.

### 2.4. Biocompatibility of AMPEC4

#### 2.4.1. Viability/Cytotoxicity

**Figure 5:**
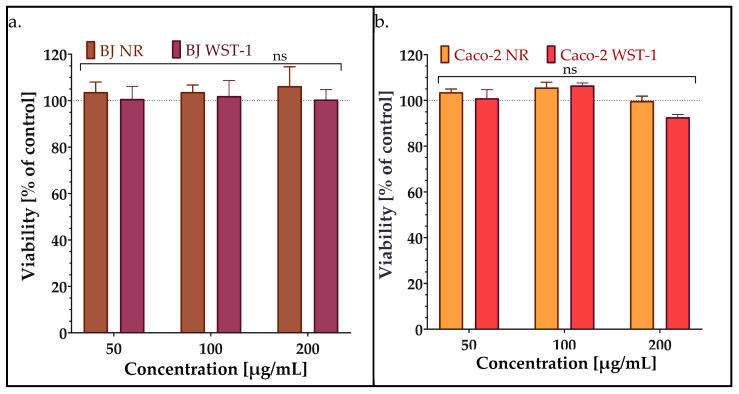
Viability of BJ (**a**) and Caco-2 (**b**) after 48 h treatment with AMPEC4 (50, 100 and 200 µg/mL); no statistical significance (ns) was observed between groups treated with different concentrations and between assays; the dashed line indicates the viability of the untreated peptide control.

The development of safe antibacterial compounds is important for the effective treatment of different pathogens. Of the plethora of inhibitory compounds discovered, only a few are able to enter the market. Many of them are quite effective in killing pathogens but are cytotoxic to eukaryotic cells [26]. Therefore, to evaluate the influence of synthesized AMPEC4 peptide in the ½ MIC, MIC and 2MIC concentrations (50, 100 and 200 µg/mL), the human normal fibroblasts and human colorectal adenocarcinoma, respectively, BJ and Caco-2 cell lines, were used. Two different assays, neutral red (NR) and WST-1, were used to assess cytotoxicity because they evaluate cell viability through distinct mechanisms, providing complementary information. The viability of both BJ and Caco-2 cell lines was not affected by the 48 h incubation with different concentrations of AMPEC4 (Figure 5).

#### 2.4.2. Hemolysis Assay

**Figure 6:**
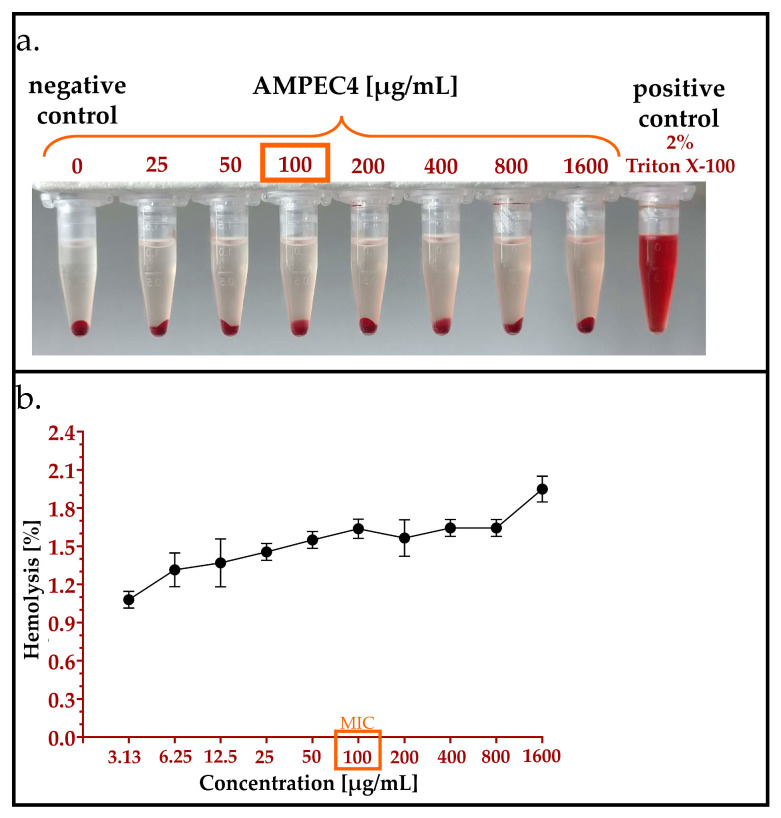
Effect of different concentrations of AMPEC4 (3.13–1600 µg/mL) after 1 h incubation with sRBCs in 37 °C (**a**) and percentage of hemolysis of sRBC vs. peptide concentration (**b**).

An experimental method was used to determine the toxicity of AMPEC4 peptide towards sheep red blood cells (sRBCs). The hemolysis assay revealed that the incubation of sRBCs with AMPEC4, even at a concentration of 1600 µg/mL, resulted in the destruction of approximately 2% of these non-nucleated cells (Figure 6a,b). Although the observed hemolysis affected a maximum of 2% of sRBCs, a dose-dependent response was evident. This observation indicates that AMPEC4 exhibits non-hemolytic properties and supports its biocompatibility.

#### 2.4.3. Genotoxicity Assay

**Figure 7:**
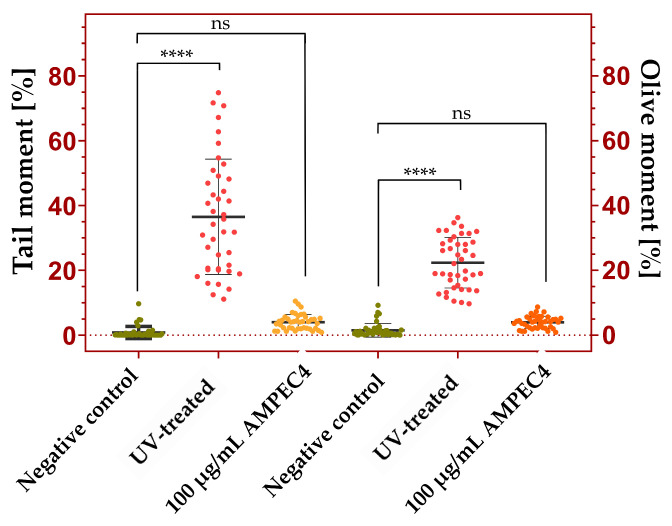
Biocompatibility of AMPEC4 on BJ cells after treatment with 100 µg/mL peptide and UV for 1 h. One-way ANOVA followed by Dunnett’s multiple comparisons test was performed using GraphPad Prism version 8.1 for Windows, GraphPad Software, Boston, MA, USA, www.graphpad.com (**** *p* < 0.0001, no statistical significance (ns) between groups).

DNA damage is assessed based on the amount of DNA in the comet tail, with key parameters serving as reliable indicators: tail moment (tail intensity × tail length) and Olive moment (%tail DNA × distance between head and tail centers) (Figure 7) [37,38]. In contrast to UV exposed cells (λ = 254 nm, 200 mW/cm^2^), both parameters indicate no genotoxicity of 100 µg/mL AMPEC4 after 1 h treatment.

#### 2.4.4. In Silico Binding Evaluation

In order to evaluate potential molecular mechanisms of AMPEC4 biological activity, a total of 27 molecular targets related to fibroblast proliferation were selected. Targets can be divided into three distinct groups: integrins, receptors and fibroblast growth factors. Respective UniProt database codes and links to corresponding entries in UniProt and AlphaFold databases are included in Table A1 [39].

To evaluate potential binding between selected targets and AMPEC4, peptide homology models generated by the AlphaFold project were analysed. Unfortunately, in many cases, available models were of low quality, with significant parts of the protein lacking defined structural features. Despite this obstacle for each of the targets, we determined the volume for the putative cavities. Targets with cavity volume exceeding 2000 Å^3^, which represents the calculated volume of the AMPEC4 monomer, were selected and used for further analysis (Table 2).

**Figure 8:**
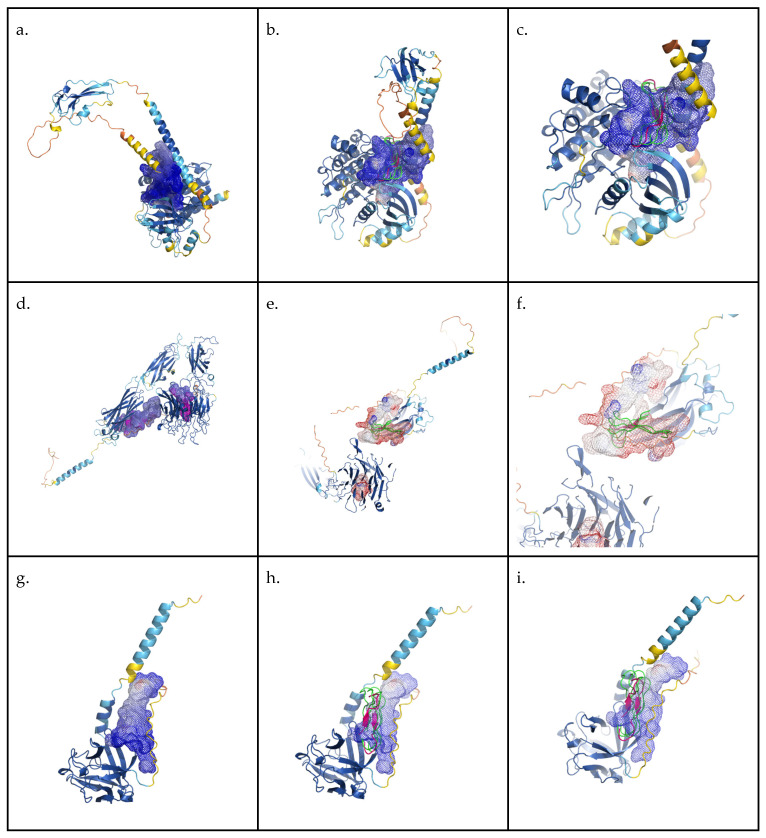
Graphical representation of cavity prediction and docking simulation results. Panels (**a**–**c**) present results for P36894 bone morphogenetic protein receptor type-1A, (**d**–**f**) P06756 integrin alpha-V, and (**g**–**i**) O76093 fibroblast growth factor 18. The secondary structure of the AlphaFold models (access date: 18 March 2025) is colored according to the pLDDT score (red (low confidence)–yellow–blue (high confidence)) [39]. Mesh represents detected cavities colored according to the calculated Coulomb potential and is generated by the CavitOmiX (v. 1.0, https://innophore.com/) plugin. AMPEC4 in purple and cartoon represents top-scoring docking pose and in green and ribbon represents the alternative top 3 binding poses. The figure was prepared with PyMol open-source version 3.1.0.

Based on the manual inspection of the obtained models, as well as the cavity prediction results, single molecular targets from each category were selected for AMPEC4 docking evaluation: P36894 bone morphogenetic protein receptor type-1A, P06756 integrin alpha-V, O76093 fibroblast growth factor 18. Docking simulations were performed using the HADDOCK 2.4 web server. The results are presented in Table 3 and Figure 8.

## 3. Discussion

The AMPEC4 synthetic peptide was derived from a structural fragment of cytotoxin 5, a 3FTx protein originating from *Naja ashei* venom, previously documented for its antibacterial properties [30]. Cytotoxin 5 (3SA5_NAJMO, P25517) is a member of the 3FTx protein family, which we previously identified in the F2 fraction obtained through the chromatographic separation of *Naja ashei* venom in our earlier analysis [30]. The venom of this species is typical for elapid snakes and consists mostly of cytotoxic 3FTxs and phospholipases A_2_ [43,44]. This fraction demonstrated significant antibacterial activity against *Staphylococcus epidermidis*, exhibiting a synergistic effect with ampicillin and tetracycline while substantially inhibiting biofilm formation. However, the precise mechanism by which the components of this fraction exert their antibacterial effects remains unclear, largely due to the complexity of the mixture. The F2 fraction consists of six major protein groups present in quantities exceeding 5%, along with numerous other proteins in trace amounts [30]. Notably, the fraction includes a protein homologous to cytotoxin 5, with its amino acid sequence serving as the basis for the design of the AMPEC4 peptide. Proteins belonging to the three-finger toxin (3FTx) family, of which this protein is a member, accounted for 15% of the total protein content in the F2 fraction and are known for their documented antibacterial properties. Our findings clearly indicate that AMPEC4 “inherited” the antibacterial activity from 3FTxs but did not show their cytotoxicity toward human cells and even has a positive effect on them by stimulating cell migration. This means that the designed peptide has dual activity, antibacterial and stimulating human fibroblasts, which are desirable in the wound healing process.

Cardiotoxins derived from the venoms of *Naja nigricollis* [14] and *Naja naja atra* [15] have been shown to inhibit the growth of both Gram-positive (*Staphylococcus aureus*) and Gram-negative (*Escherichia coli*) bacteria. These toxins function by inducing membrane permeabilization, leading to bacterial cell death through the disruption of the membrane’s structural integrity [14,15]. The confirmed penetration of propidium iodide into the bacterial cells during analysis indicates that the AMPEC4 peptide likely induces similar membrane damage and disruption. It is hypothesized that the peptide sequence responsible for these effects may be derived from a portion of the cardiotoxin involved in binding to membrane components [45]. This hypothesis is further supported by prior studies on interactions between 3FTx proteins from *Naja ashei* venom and membrane phospholipids [46,47]. In both cases, the venom fractions contained a protein homologous to cytotoxin 5, which includes a fragment of its sequence that was utilized in the design of AMPEC4. This suggests that the peptide may interact with bacterial membrane components, compromising their structural integrity.

To confirm the hypothesis that AMPEC4 enhances cell membrane permeability, rhodamine-labeled peptide and propidium iodide (PI) were used. The results clearly demonstrated that AMPEC4 interacts with the bacterial membrane, facilitating PI penetration into previously impermeable cells. Additionally, the concentration-dependent effect of AMPEC4 on bacterial membrane integrity indicates that at ½ MIC, the peptide induces transient membrane disruptions, whereas at MIC, it causes permanent disorganization, ultimately leading to rhodamine-labeled AMPEC4 leakage from the cells. The observed decreasing intensity of PI fluorescence after treatment with 50 µg/mL peptide (½ MIC) may be caused by the activity of efflux pumps [48,49], which can effectively extrude an array of substrates, including common antibiotics, dyes, and biocides [49]. However, this mechanism is only a hypothesis and must be supported by additional studies, especially with the use of broad-spectrum (e.g., carbonyl cyanide 3-chlorophenylhydrazone, CCCP [50]), as well as specific (e.g., verapamil as ATP-binding cassette, ABC, inhibitor [51]) efflux pump inhibitors.

Interestingly, cytotoxins, as the name implies, are highly toxic to animal cells and are sometimes referred to as cardiotoxins due to their direct toxicity to cardiomyocytes [52]. At high concentrations, venoms rich in cytotoxins, such as cobra venom, can induce apoptosis in cells, resulting in tissue necrosis, as observed clinically [53]. At lower concentrations, these toxins inhibit cell migration and proliferation [54]. The selective cytotoxicity of these proteins toward cancer cells has also garnered attention for the development of anti-cancer therapies based on venom-derived cytotoxins [55]. The AMPEC4 peptide corresponds to the N-terminal fragment of cytotoxin 5, encompassing approximately one-third of the full protein sequence. This length is sufficient to form one of the characteristic “fingers” of 3FTx proteins, which is likely responsible for its membrane-disrupting activity in bacterial cells (Figure 1). However, this structure appears to be inadequate for inducing cytotoxicity in human cells. In contrast, analysis reveals that the AMPEC4 peptide promotes the proliferation and migration of fibroblasts without affecting their viability. The precise mechanism underlying this effect on fibroblast development remains unclear, though it is certain that it does not involve the FGF2-based signaling pathway or vimentin stimulation (data not presented). The performed docking simulation suggests that AMPEC4 can bind to selected target cavities. Top-scoring binding poses are concentrated within a single cavity for each of the tested proteins with comparable orientation (Figure 7).

Biocompatibility determines a drug’s ability to interact with biological systems without causing toxicity or adverse effects, ensuring both safety and efficacy. Evaluating biocompatibility helps optimize drug formulations and delivery, minimizing harm to surrounding tissues. The NR assay assesses lysosomal integrity by measuring the uptake and retention of neutral red dye, detecting early cellular damage [56]. WST-1a evaluates metabolic activity by measuring mitochondrial dehydrogenase activity, correlating with cell viability [57]. Using both assays provides a comprehensive cytotoxicity assessment, distinguishing effects on lysosomal function and metabolism. Both tests indicated that AMPEC4 does not impact the viability of eukaryotic cells, including both normal and cancerous types. These findings strongly suggest that the designed peptide exhibits high cellular selectivity, offering a promising safety profile for potential use in treating bacterial infections without causing harm to host cells at the infection site.

Genotoxicity assessment is also crucial in drug design because it helps identify potential risks of DNA damage that could lead to mutations, cancer, or other genetic disorders [37]. The comparison of the Olive moment and Tail moment obtained from the comet assay reveals differences in their sensitivity to DNA damage. The Tail moment, calculated as the product of tail intensity and tail length, provides a general measure of DNA fragmentation and migration. In contrast, the Olive moment, which incorporates the distance between the centers of the comet head and tail, offers a more refined assessment of DNA damage distribution. While both parameters correlate with the extent of DNA strand breaks, the Olive moment may be more sensitive in detecting subtle variations in damage, particularly in samples with heterogeneous DNA fragmentation [37,38]. AMPEC4 does not induce genotoxicity in the analyzed cells. The results from the comet assay were indistinguishable from those of the control cells, further supporting the conclusion that the designed peptide is a potentially safe antibacterial agent with no detrimental effects on human cell integrity.

A huge consumption of broad-spectrum antibacterial drugs has resulted in the emergence of many multidrug-resistant strains [58], and much attention is currently devoted to developing new compounds. The synthesized peptide, exhibiting both antibacterial and wound-healing capabilities derived from a snake venom, presents a compelling avenue for the development of innovative therapeutics targeting infection and tissue repair.

## 4. Materials and Methods

### 4.1. Chemicals

Chemicals used for microbiological assays: Mueller Hinton Broth (MHB), Mueller Hinton Agar (MHA), ampicillin, chloramphenicol, neutral red solution (0.33%, 3-Amino-7-dimethylamino-2-methylphenazine hydrochloride), and PBS (phosphate buffered saline), MTT (3-(4,5-dimethyl-2-thiazolyl)-2,5-diphenyl-2H-tetrazolium bromide) were obtained from Merck (Darmstadt, Germany).

Chemicals used for cell culture were purchased from the ATCC (Manassas, VA, USA): normal skin fibroblasts (BJ CRL-2522), human colorectal adenocarcinoma (Caco-2, HTB-37), Eagle’s Minimum Essential Medium (EMEM, 30-2003), fetal bovine serum (FBS, 30-2020), Trypsin-EDTA solution (30-2101), Dulbecco’s Phosphate Buffered Saline (D-PBS, 1X, 30-2200), and Penicillin (50 U/mL)–Streptomycin (50 µg/mL) Solution (Pen-Strep, 30-2300).

Chemicals used for cell assays: normal-melting-point agarose (NMA) and low melting point agarose (LMA) were purchased from Merck (Darmstadt, Germany); sodium chloride, EDTA, Tris base, Triton X-100, sodium acetate, ammonium acetate and ethyl alcohol (96%) and Sheep Blood Defibrinated were obtained from Pol-Aura (Zabrze, Poland); Hoechst 33342 was obtained from Thermo Fisher Scientific, San Diego, CA, USA; and propidium iodide and cell proliferation reagent WST-1 (abcam, Cambridge, UK) were also used.

Chemicals used for synthesis: Fmoc protected amino acids (Carbolution Chemicals GmbH, Ingbert, Germany), Fmoc-Rink Amide resin (Carbolution Chemicals GmbH, Ingbert, Germany), *N*,*N*-dimethylformamide (POCH, Avantor, Gliwice, Poland), piperidine (Iris Biotech GmbH, Marktredwitz, Germany), DIC (*N*,*N’*-diisopropylcarbodiimide; Carbolution Chemicals GmbH, Ingbert, Germany), OxymaPure (Iris Biotech GmbH, Marktredwitz, Germany), trifluoroacetic acid (Apollo Scientific, Denton, UK), rhodamine (Sigma, Schnelldorf, Germany), triisopropylsilane (Iris Biotech GmbH, Marktredwitz, Germany), phenol (Merck, Darmstadt, Germany), diethyl ether (POCH, Avantor, Gliwice, Poland), and acetonitryle (POCH, Avantor, Gliwice, Poland).

### 4.2. AMPEC4 Peptide Design

The peptide AMPEC4 was designed based on a tryptic peptide from cytotoxin 5 (P25517) by removing three C-terminal amino acids. The physicochemical properties of the designed peptide were evaluated using free web servers (https://aps.unmc.edu/ (accessed on 20 March 2025), https://www.bachem.com/knowledge-center/peptide-calculator/ (accessed on 20 March 2025)).

### 4.3. Structure Analysis and Visualization

Models of investigated molecular targets were obtained from the PDB database (https://www.ebi.ac.uk/pdbe/, access date 18 March 2025) or the AlphaFold database (https://alphafold.ebi.ac.uk/, access date 18 March 2025).

The volume of the AMPEC4 was calculated with the ProteinVolume online calculator (https://gmlab.bio.rpi.edu/PVolume.php, access date 18 March 2025) with default parameters [59].

Figures were prepared with PyMOL open source version 3.1.0 [42]. Cavities were calculated using CavitOmiX (v. 1.0, 2022, Innophore GmbH, Graz, Austria). The corresponding hydrophobicity module of the program VASCo was used to analyze the hydrophobicity of the cavities. The cavities were calculated using a modified LIGSITE algorithm [40,41]. Active residues for the configuration of the HADDOCK docking experiment were identified with the PyMol CavitOmiX plugin for each of the selected proteins and cavities meeting cutoff criteria. The residues lining cavities, as well as all residues of AMPEC4, were treated as active residues, and the remaining parameters were kept at default values.

### 4.4. AMPEC4 Peptide Synthesis

The AMPEC4 peptide (LKCKKLIPLFSKTCP-NH_2_) was synthetized automatically on a microwave Liberty Blue™ Automated Microwave Peptide Synthesizer (CEM Corporation, Mathews, NC, USA) by the solid-phase Fmoc/tBu methodology, where polystyrene resin modified by the Fmoc-Rink Amide (loading 0.63 mmol/g) linker was used as a solid support. Before synthesis resin was swelled with *N*,*N*-dimethylformamide (DMF) for 5 min at RT. The deprotection of the Fmoc group was performed with a 20% (*v/v*) piperidine solution in DMF. Couplings were performed with an equimolar mixture of Fmoc-AA-OH, *N*,*N*’-diisopropylcarbodiimide (DIC) and Oxyma Pure in fivefold excess based on the resin. Each cycle consisted of a deprotection step, the washing of the resin and the subsequent coupling of Fmoc-protected amino acid. Deprotection was performed in two stages, the first at 75 °C (180 W) for 15 s and the second at 90 °C (45 W) for 55 s. Then, the resin was washed four times with DMF. Coupling reactions were carried out in two stages, the first at 75 °C (155 W) for 15 s and the second at 90 °C (30 W) for 110 s. To provide sufficient mixing during coupling and deprotection, the vessel with resin and reagents was bubbled with nitrogen (repeating sequence—bubble on for 2 s, off for 3 s). The elongation of the peptide chain was carried out in consecutive cycles of deprotection and coupling. Rhodamine B was coupled with an equimolar mixture of rhodamine B, DIC and Oxyma Pure in 3-fold excess in darkness at room temperature over 1.5 h with constant shaking. The peptide was cleaved from the resin using the mixture of trifluoroacetic acid (TFA), triisopropylsilane (TIS), ethane-1,2-dithiol (EDT), phenol and deionized water (90:2.5:2.5:2.5:2.5) for 1.5 h with agitation in darkness. Then, the product was precipitated with cooled diethyl ether and lyophilized. Purification was carried out by RP-HPLC on a Phenomenex Gemini-NX C18 column (21.2 × 100 mm, 5.0-μm particle size, 110-Å pore size). The eluents used were water and acetonitrile containing 0.1% (*v/v*) of TFA. A linear 10–70% acetonitrile gradient in deionized water over 90 min was used, and the mobile phase flow rate was 20.0 mL/min. The purity and identity of the peptides were confirmed with LC-MS analysis. The RP-HPLC system was used—Waters Alliance e2695 system with Waters 2998 PDA and Acquity QDA detectors (software—Empower^®^3, Waters, Milford, MA, USA). All analyses were carried out on a Waters XBridge™ Shield RP-18 column (3.0 × 100 mm, 3.5 µm particle size, 130 Å pore size). Samples (10 µL) were analyzed with a linear 10–90% acetonitrile gradient in deionized water over 15 min at 25.0 ± 0.1 °C. The mobile phase flow rate was 0.5 mL/min. Both eluents contained 0.1% (*v/v*) of formic acid. Mass analysis and UV detection at 214 nm were used. Pure fractions (>95%, by HPLC analysis) were collected and lyophilized. The synthesis yield was 46% for AMPEC4 and 32% for rhdAMPEC4.

### 4.5. Antimicrobial Activity Testing

The potential in vitro antibacterial activity of synthesized peptide was investigated against certified *Escherichia coli* ATCC 10536 from a collection of the Department of Biotechnology and Bioinformatics, Faculty of Chemistry, Rzeszow University of Technology, and clinical *Escherichia coli* 325 (human semen culture), 665 and 672 (human urine cultures) strains, which were obtained from the Department of Medical Laboratory Diagnostics of the Provincial Specialist Hospital in Rzeszow. To ensure normal growth patterns, all bacterial strains were grown from frozen stocks and subcultured at 37 °C in a New Brunswick Innova 40 Shaker (Eppendorf AG, Hamburg, Germany) at least twice before use in experiments. The initial 24 h bacterial culture was the same for all experiments and was prepared by adjustment to the 0.5 McFarland standard (10^8^ CFU/mL) using a Varioskan™ LUX multimode microplate reader (Thermo Scientific^TM^, Waltham, MA, USA), λ = 600 nm.

#### 4.5.1. Determination of Minimum Inhibitory Concentration (MIC)

The antibacterial activity of the AMPEC4 was evaluated by obtaining the minimum inhibitory concentrations (MIC, µg/mL) using the micro-broth dilution method in MHB, as previously described [60,61]. The minimum inhibitory concentration (MIC) is the lowest concentration of antibacterial agent at which the growth of bacteria is completely inhibited. Thus, the lower the MIC value, the better the antibacterial properties of a given compound [30]. AMPEC4 peptide was tested as an antibacterial agent in the range of concentrations from 0.78 to 200 µg/mL prepared by the microdilution method on 96-well plates. An evaluation of the antibiotic susceptibility of each bacterial strain to ampicillin and chloramphenicol was also performed by the microdilution method (concentration range from 0.78 to 100 µg/mL). Working bacterial cultures were diluted to a final density of 10^5^ CFU and added to each well of prepared plates, except for those used as media sterility. After 24 h of incubation at 37 °C, the optical density measurement at 600 nm was performed using the Varioskan™ LUX multimode microplate reader. The medium without antibacterial agents and wells with no bacterial cultures added served, respectively, as a control of bacterial growth and a solvent control. All experiments were carried out in three technical and biological repetitions to ensure the reproducibility of the results.

#### 4.5.2. Inhibition of Biofilm Formation

To evaluate the ability of the synthesized peptide to prevent biofilm formation by reference and clinical *E. coli* strains, the MTT assay was performed. The MTT staining assay was conducted to measure viable cells in the biofilms, as MTT can react with dehydrogenase enzymes in viable cells to form blue-violet formazan. Overnight cultures of tested strains were diluted into approximately 10^5^ CFU/mL with MHB and treated with various concentrations of testing peptides in 96-well cell culture plates. After 24 h of incubation at 37 °C, the plates were rinsed twice with 1×PBS to remove non-adhering and planktonic cells, MTT solution (200 µg/mL, 1× PBS) was added to cells, and plates were incubated at 37 °C in the dark for 2 h. Formazan crystals formed in viable cells were then dissolved by the addition of DMSO, and the color intensity of the resulting solution was measured spectrophotometrically at 570 nm using the Varioskan™ LUX multimode microplate reader. The assay was repeated at least three times. The percentage of biofilm formation (BF) was determined by the formula: %BF = (Control OD570 nm/Test OD570 nm) × 100%. The culture without added peptides was used as a control, and the wells containing the culture medium alone were used as blanks.

#### 4.5.3. Biofilm Eradication

The efficacy of AMPEC4 in eliminating pre-formed biofilms was evaluated using the MTT assay on biofilm-producing bacterial strains *E. coli* ATCC 10536 and *E. coli* 672. Bacterial cultures were prepared according to the methodology outlined in Section 4.5.2. Prior to the addition of AMPEC4, biofilms were established by incubating bacterial cultures for 24 h at 37 °C in 96-well plates, followed by the removal of planktonic bacteria, leaving the adhered biofilm. Subsequently, AMPEC4 solutions prepared in MHB at concentrations ranging from 6.25 to 200 µg/mL were introduced. Following an additional 24 h incubation period at 37 °C, the MTT assay was conducted, and the results were processed as previously described.

#### 4.5.4. Bacterial Membrane Permeabilization Assay

The cell membrane permeability of the reference *E. coli* strain (ATCC 10536) after treatment with rhdAMPEC4 peptide at ½ MIC (50 µg/mL) and MIC (100 µg/mL) concentrations was assessed using propidium iodide (PI). The overnight culture was adjusted to 10⁸ CFU/mL, then treated with AMPEC4 to achieve the indicated final concentrations and also with PI solution (final concentration 2.5 µg/mL). After the incubation of the bacteria/peptide/PI mixture at 37 °C in the dark for 5, 10, 15, 30, and 45 min, the samples were centrifuged for 2 min at 4000 rpm, and 300 µL of 1 × PBS was added. Following the creation of a uniform bacterial cell suspension, 100 µL of each sample, in triplicate, was transferred to a black plate with an optical glass bottom (Thermo Fisher Scientific, CA, USA), and fluorescence intensity was measured at λex = 535 nm/λem = 617 nm for PI and λex = 352 nm/λem = 455 nm for rhdAMPEC4. This experiment was performed in three biological repetitions.

### 4.6. Cell Line Studies

Normal human fibroblast (BJ) and human colorectal adenocarcinoma (Caco-2) cell lines were cultured in EMEM supplemented with 10% (*v/v*) FBS and Pen-Strep and maintained in a humidified atmosphere at 37 °C, with 5% CO_2_. The dissociation of adherent cells was performed with Trypsin-EDTA solution, and the cells were counted using the TC20 Automated Cell Counter (Bio-Rad, Hercules, CA, USA) by trypan blue staining [62]. A maximum of 10 passages was used to maintain phenotypic cell characteristics.

#### 4.6.1. Scratch Assay

**Figure 9:**
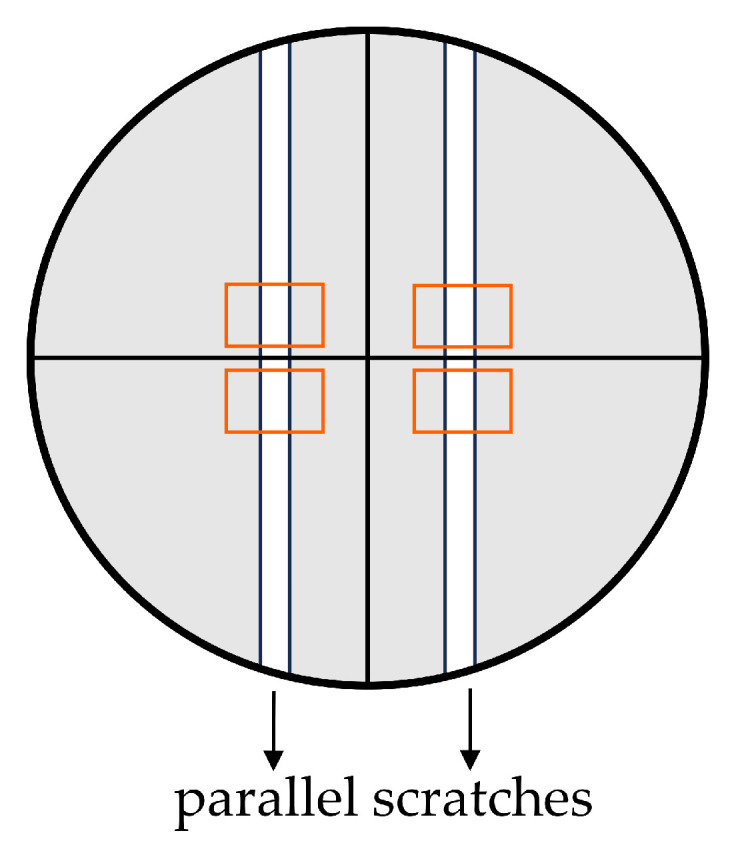
Diagram showing the preparation of scratches for performing the scratch test in a representative well of a 24-well plate: the area filled with BJ cells showing adhesion to the surface (gray areas), marking the observation site (perpendicular black lines), parallel scratches made by 200 µL pipette tips (white rectangles), and precise areas of observation of BJ cell migration (orange rectangles).

To assess the migratory capabilities of BJ cells following exposure to AMPEC4 peptide at concentrations of ½ MIC (50 µg/mL) and MIC (100 µg/mL), a scratch assay, also known as a wound healing assay, was conducted. The experimental procedure involved seeding cells at a density of 5 × 10^4^ cells/mL, followed by overnight incubation to achieve approximately 95% confluency. Subsequently, two parallel scratch lines were introduced into the confluent monolayer in each well of a 24-well plate using a sterile 200 µL pipette tip (Figure 9). Following the creation of the scratches, the cells were washed twice with D-PBS to remove detached cells. Then, 50 µg/mL and 100 µg/mL AMPEC4 in fresh EMEM containing 5% FBS, as well as medium without the tested peptide (cell migration control), was introduced. Image acquisition was performed using an Olympus IX83 inverted microscope (Olympus, Shinjuku, Japan) after 6 and 18 h after creating scratches and treatment with AMPEC4. The experiment was conducted in three technical repetitions and comprised two independent series, resulting in a total of six replicates (wells) for each condition. The acquired images were analyzed using ImageJ 1.54i software [63], and the extent of cell migration into the wound area (%) was quantified as described before [24,62].

#### 4.6.2. Biocompatibility Assay

Biocompatibility assessment, as a crucial step in drug design, ensures that the developed compounds do not cause harmful effects when administered to living organisms [64,65,66]; it was performed based on ISO 10993-1:2018 [67] and concerned the analysis of the blood compatibility and cyto- and genotoxicity of AMPEC4 peptide.

##### Viability Assay

To assess the cytotoxicity of AMPEC4 peptide in BJ and Caco-2 cell lines, WST-1 and NR assays were used. The final concentrations of AMPEC4 were 50, 100 and 200 µg/mL. The WST-1 assay measures cell viability based on the production of formazan dye, which is directly proportional to cellular metabolic activity [33]. Both cell cultures were seeded in 96-well plates at a density of 10^4^ cells per well and incubated overnight at 37 °C with 5% CO_2_. Following a 48 h treatment with different concentrations of AMPEC4, WST-1 [33] and NR [56] assays were performed as described before. Cell viability was calculated and expressed as a percentage relative to untreated control cells. All treatments were performed in triplicate across three independent experiments.

##### Hemolysis Assay

The assay was conducted according to the procedure reported by Neubauer et al. (2020) [68] and Kamysz et al. (2023) [69] with modifications. Briefly, the fresh sheep blood was rinsed very gently three times with PBS to obtain sRBCs only in PBS solution. Then, centrifugation at 800× *g* for 10 min was performed, and sRBCs were resuspended in PBS. The serial dilution of peptide (3.13–1600 µg/mL) was prepared in PBS in test tubes with conical bottoms. Then, the 8% sRBCs stock solution in PBS was added to each test tube at a 1:1 ratio to obtain 4% of sRBCs. The control wells for 0% (RBCs in PBS without AMPEC4) and 100% (RBCs in 2% Triton-X 100) hemolysis were also prepared. Afterward, prepared mixtures were incubated for 1 h at 37 °C with agitation and centrifuged at 800× *g* for 10 min at 4 °C (Sorvall ST 16R Centrifuge, Thermo Scientific, Waltham, MA, USA). After centrifugation, the supernatants were carefully transferred to a 96-well plate in three repetitions and the release of hemoglobin was measured at 540 nm with the use of a Varioskan™ LUX multimode microplate reader. The test was performed in three independent repetitions, and the percentage of hemolysis was calculated based on wells with 100% hemolysis.

##### Genotoxicity

BJ cells were used to identify potential risks of DNA damage that could lead to mutations, cancer, or other genetic disorders [38,70], as a result of AMPEC4 treatment. Fibroblasts were seeded on 90 mm cell culture Petri dishes at a density of 10^6^ cells/well and were incubated overnight at 37 °C with 5% CO_2_. Subsequently, the medium was discarded, and 50 µg/mL AMPEC4 in complete medium was added and incubated for 1 h. A positive and negative control were used, respectively, and BJ cells were treated with UV (λ = 254 nm, 200 mW/cm^2^, t = 1 h) and nontreated BJ cells. Comet assay procedure was performed as described earlier [71,72,73]. Briefly, the day before, slides were prepared by coating them with 1% of NMA and allowed to air dry and solidify overnight at RT. The next day, after treatment of BJ cells with AMPEC4 or UV for 1 h, cells were trypsinized to release them from the cell culture Petri dishes and neutralized by adding serum-containing media. Cells (10^5^ cells/mL) were mixed with molten 0.75% LMA at a ratio of 1:10 (*v/v*), immediately pipetted onto slides prepared earlier, and kept on ice for 10 min (in the dark). Slides were then incubated in a pre-chilled lysis solution (3.5 M NaCl, 100 mM EDTA, 100 mM Tris base, 1% Triton X-100, pH 10.0) on ice for 1 h and washed in pre-chilled 1× electrophoresis buffer (10× concentrated buffer was prepared by mixing 60.57 g Tris base, 204.12 g sodium acetate in 500 mL of dH_2_O, pH 9.0) on ice for 30 min, and electrophoresis was performed for 1 h at 4 °C (21 V). The excess of 1× electrophoresis buffer was drained, and the slides were kept in precipitation solution (7.5 M ammonium acetate was mixed with 95% EtOH at a ratio 1:6 (*v/v*)) for 30 min at RT, immersed in 70% EtOH, and dried for 15 min at RT. Hoechst 33342 (1 µg/mL) was used to visualize comets under IX83 Olympus fluorescence and ImageJ with the Open Comet plugin [37] to analyze comet images. The outliers were manually removed from the data, and Olive and Tail moments were calculated and graphically presented using GraphPad Prism version 8.0.1 (GraphPad Software, Boston, MA, USA, www.graphpad.com).

### 4.7. Docking Simulations

Simulations were performed with HADDOCK (High-Ambiguity-Driven protein–protein DOCKing) web server version 2.4, offering an information-driven flexible docking approach for the modeling of biomolecular complexes [74]. The domain representing AMPEC4 was extracted from the P25517 AlphaFold homology model. The molecular targets structures were obtained from AlphaFold database and used without modifications.

A list of amino acids representing a putative binding site was obtained as a part of the cavity detection process.

Docking was performed with default simulation parameters. Results for the top-scoring cluster are reported.

### 4.8. Statistical Analysis

All the experiments were carried out in three technical repetitions and 2-3 biological repetitions, and the results indicate the mean ± standard deviation (SD). Statistical significance between treatment groups was evaluated using the one-way ANOVA test followed by Tukey’s or Dunnett’s multiple comparisons A value of *p* < 0.05 was considered statistically significant. All data processing in this study was carried out using GraphPad Prism version 8.0.1 (GraphPad Software, Boston, MA, USA, www.graphpad.com).

## 5. Conclusions

Venom-derived peptides offer a promising avenue for identifying novel antibacterial compounds in the era of multidrug-resistant bacteria. AMPEC4 is a newly designed synthetic peptide with dual functionality, exhibiting both antibacterial activity and the ability to promote fibroblast migration, making it a promising candidate for proctological wound healing. The selective antibacterial activity and lack of cytotoxicity of AMPEC4 make it a strong candidate for further development as a therapeutic agent against bacterial infections. Future studies should focus on optimizing its stability, bioavailability, and in vivo efficacy to fully explore its potential in clinical applications.

## Figures and Tables

**Table 1:** Minimum inhibitory concentrations (µg/mL) of studied peptide and antibiotics against reference and clinical *Escherichia coli* strains.

Bacterial Strains	Reference	Clinical		
	ATCC 10536	325	665	672
AMPEC4	100.0	200.0	100.0	200.0
Ampicillin	31.3	62.5	NAA ^1^	NAA ^1^
Ciprofloxacin	0.5	0.25	0.25	0.5
Chloramphenicol	1.0	3.9	3.9	3.9

^1^ NAA—no antibacterial activity in the tested concentration range (from 0.01 to 200 µg/mL).

**Table 2:** For selected molecular targets: integrins (*), receptors (^#^) and fibroblast growth factors (^$^), a cavity detection was performed with PyMol (v. 3.1.0, open source version) plugin CavitOmiX (v. 1.0, https://innophore.com/) [40,41,42]. The significance cutoff for detected cavities was set at 2000 Å^3^, which approximates the calculated volume of an AMPEC4 monomer. Targets with multiple putative cavities are listed as separate entries. Entries are sorted according to calculated cavity volume.

Molecular Target	Cavity Volume (Å^3^)
P21802 ^#^	26,172
P11362 ^#^	23,015
P22455 ^#^	21,638
P06756 *	18,607
P22607 ^#^	15,623
P05106 *	11,377
P42702 ^#^	7466
P22455 ^#^	7209
P21802 ^#^	5469
Q13873 ^#^	4800
P22607 ^#^	4350
P36894 ^#^	3773
P06756 *	3411
Q13873 ^#^	2976
Q13873 ^#^	2460
Q13873 ^#^	2376
O76093 ^$^	2371
Q13873 ^#^	2354
P42702 ^#^	2194
P22607 ^#^	2184
Q13873 ^#^	2103

**Table 3:** Docking simulation results. HADDOCK score, for the top cluster, and Z-score, indicating how many standard deviations from the average this cluster is located in terms of score (the more negative the better), are reported.

UniProt ID	Cavity Volume (Å^3^)	HADDOCK Score	Z-Score
P36894	3773	−51.2 ± 8.9	−1.7
P06756	3411	−89.9 ± 11.0	−1.7
O76093	2371	−95.7 ± 10.1	−1.6

## Data Availability

Data are contained within the article.

## Appendix A

**Table A1:** Molecular targets selected for analysis. All the reported data were obtained from UniProt databse (https://www.uniprot.org/, access date 18 March 2025).

Molecular Target	Database Links
Integrins	
Integrin alpha-V	https://www.uniprot.org/uniprotkb/P06756/entry https://alphafold.ebi.ac.uk/entry/P06756
Integrin beta-3	https://www.uniprot.org/uniprotkb/P05106/entry https://alphafold.ebi.ac.uk/entry/P05106
Receptors	
Fibroblast growth factor receptor 2	https://www.uniprot.org/uniprotkb/P21802/entry https://alphafold.ebi.ac.uk/entry/P21802
Fibroblast growth factor receptor 3	https://www.uniprot.org/uniprotkb/P22607/entry https://alphafold.ebi.ac.uk/entry/P22607
Fibroblast growth factor receptor 4	https://www.uniprot.org/uniprotkb/P22455/entry https://alphafold.ebi.ac.uk/entry/P22455
Bone morphogenetic protein receptor type-1A	https://www.uniprot.org/uniprotkb/P36894/entry https://alphafold.ebi.ac.uk/entry/P36894
Bone morphogenetic protein receptor type-2	https://www.uniprot.org/uniprotkb/Q13873/entry https://alphafold.ebi.ac.uk/entry/Q13873
Leukemia inhibitory factor receptor	https://www.uniprot.org/uniprotkb/P42702/entry https://alphafold.ebi.ac.uk/entry/P42702
Fibroblast growth factor receptor 1	https://www.uniprot.org/uniprotkb/P11362/entry https://alphafold.ebi.ac.uk/entry/P11362
Fibroblast growth factors	
Fibroblast growth factor 2	https://www.uniprot.org/uniprotkb/P09038/entry https://alphafold.ebi.ac.uk/entry/P09038
Fibroblast growth factor 14	https://www.uniprot.org/uniprotkb/Q92915/entry https://alphafold.ebi.ac.uk/entry/Q92915
Fibroblast growth factor 1	https://www.uniprot.org/uniprotkb/P05230/entry https://alphafold.ebi.ac.uk/entry/P05230
Fibroblast growth factor 10	https://www.uniprot.org/uniprotkb/O15520/entry https://alphafold.ebi.ac.uk/entry/O15520
Fibroblast growth factor 16	https://www.uniprot.org/uniprotkb/O43320/entry https://alphafold.ebi.ac.uk/entry/O43320
Fibroblast growth factor 20	https://www.uniprot.org/uniprotkb/Q9NP95/entry https://alphafold.ebi.ac.uk/entry/Q9NP95
Fibroblast growth factor 12	https://www.uniprot.org/uniprotkb/P61328/entry https://alphafold.ebi.ac.uk/entry/P61328
Fibroblast growth factor 23	https://www.uniprot.org/uniprotkb/Q9GZV9/entry https://alphafold.ebi.ac.uk/entry/Q9GZV9
Fibroblast growth factor 13	https://www.uniprot.org/uniprotkb/Q92913/entry https://alphafold.ebi.ac.uk/entry/Q92913
Fibroblast growth factor 7	https://www.uniprot.org/uniprotkb/P21781/entry https://alphafold.ebi.ac.uk/entry/P21781
Fibroblast growth factor 19	https://www.uniprot.org/uniprotkb/O95750/entry https://alphafold.ebi.ac.uk/entry/O95750
Fibroblast growth factor 21	https://www.uniprot.org/uniprotkb/Q9NSA1/entry https://alphafold.ebi.ac.uk/entry/Q9NSA1
Fibroblast growth factor 17	https://www.uniprot.org/uniprotkb/O60258/entry https://alphafold.ebi.ac.uk/entry/O60258
Fibroblast growth factor 18	https://www.uniprot.org/uniprotkb/O76093/entry https://alphafold.ebi.ac.uk/entryO76093/
Fibroblast growth factor 5	https://www.uniprot.org/uniprotkb/P12034/entry https://alphafold.ebi.ac.uk/entry/P12034
Fibroblast growth factor 8	https://www.uniprot.org/uniprotkb/P55075/entry https://alphafold.ebi.ac.uk/entry/P55075
Fibroblast growth factor 9	https://www.uniprot.org/uniprotkb/P31371/entry https://alphafold.ebi.ac.uk/entry/P31371
Fibroblast growth factor 3	https://www.uniprot.org/uniprotkb/P11487/entry https://alphafold.ebi.ac.uk/entry/P11487
